# Yellow Rust Epidemics Worldwide Were Caused by Pathogen Races from Divergent Genetic Lineages

**DOI:** 10.3389/fpls.2017.01057

**Published:** 2017-06-20

**Authors:** Sajid Ali, Julian Rodriguez-Algaba, Tine Thach, Chris K. Sørensen, Jens G. Hansen, Poul Lassen, Kumarse Nazari, David P. Hodson, Annemarie F. Justesen, Mogens S. Hovmøller

**Affiliations:** ^1^Department of Agroecology, Global Rust Reference Centre, Aarhus UniversitySlagelse, Denmark; ^2^International Center for Agricultural Research in the Dry Areas, Regional Cereal Rust Research Centre, Aegean Agricultural Research Instituteİzmir, Turkey; ^3^International Maize and Wheat Improvement Center, CIMMYTAddis Ababa, Ethiopia

**Keywords:** *Puccinia striiformis*, virulence phenotyping, resistance deployment, wheat, geographical regions

## Abstract

We investigated whether the recent worldwide epidemics of wheat yellow rust were driven by races of few clonal lineage(s) or populations of divergent races. Race phenotyping of 887 genetically diverse *Puccinia striiformis* isolates sampled in 35 countries during 2009–2015 revealed that these epidemics were often driven by races from few but highly divergent genetic lineages. *PstS1* was predominant in North America; *PstS2* in West Asia and North Africa; and both *PstS1* and *PstS2* in East Africa. *PstS4* was prevalent in Northern Europe on triticale; *PstS5* and *PstS9* were prevalent in Central Asia; whereas *PstS6* was prevalent in epidemics in East Africa. *PstS7, PstS8* and *PstS10* represented three genetic lineages prevalent in Europe. Races from other lineages were in low frequencies. Virulence to *Yr9* and *Yr27* was common in epidemics in Africa and Asia, while virulence to *Yr17* and *Yr32* were prevalent in Europe, corresponding to widely deployed resistance genes. The highest diversity was observed in South Asian populations, where frequent recombination has been reported, and no particular race was predominant in this area. The results are discussed in light of the role of invasions in shaping pathogen population across geographical regions. The results emphasized the lack of predictability of emergence of new races with high epidemic potential, which stresses the need for additional investments in population biology and surveillance activities of pathogens on global food crops, and assessments of disease vulnerability of host varieties prior to their deployment at larger scales.

## Introduction

Crop pathogens with worldwide prevalence and potential for long distance migration and invasions into new areas may pose a serious threat to food security regionally or globally (Brown and Hovmøller, 2002; Dean et al., 2012; Beddow et al., 2015). Crops like wheat, which are cultivated worldwide across diverse agro-ecological zones, provide a vast niche for their pathogens at local, regional, and continental scales (von Broembsen, 1989; Brasier and Buck, 2001). Wheat pathogens have been controlled to a large extent via ongoing and large-scale breeding efforts to improve disease resistance, which is economical, environment friendly and sometimes the only available option (Singh et al., 2016). Successful deployment of resistant crop varieties at larger scales and in different regions would, however, require better understanding of pathogen diversity for virulence across regions (Brown and Hovmøller, 2002). Large scale deployment of host varieties with narrow genetic background for disease resistance have been reported to cause the acquisition of virulence at regional and continental scales (Hovmøller et al., 2002; Singh et al., 2004; Chen, 2005; Kolmer, 2005; Wellings, 2007). This is particularly the case for biotrophic fungal pathogens, which depend on the living host for both on-season and off-season survival, and the host resistance thereby induces strong selection favoring virulence mutants of the pathogen (Hovmøller et al., 1997; McDonald and Linde, 2002; Gladieux et al., 2011).

The rust pathogens of wheat (*Triticum aestivum*) and triticale (*Triticoseale*) are among the most important crop pathogens causing a continuous threat to crop production (Singh et al., 2008; Dean et al., 2012). Three rust species infect wheat and are distributed globally, i.e., yellow (stripe) rust caused by *Puccinia striiformis* (Liu and Hambleton, 2010), leaf (brown) rust caused by *P. triticina* (Goyeau et al., 2006; Bolton et al., 2008) and stem (black) rust caused by *P. graminis* f.sp. *tritici* (Leonard and Szabo, 2005; Singh et al., 2008; Berlin et al., 2013). All the three rusts have been shown to cause huge losses in different areas, years and environments favoring disease epidemics (Dean et al., 2012; Pardey et al., 2013; Beddow et al., 2015; Singh et al., 2016). Nonetheless yellow rust in particular has been reported as an increasing problem (Hovmøller et al., 2010) with repeated cases of worldwide invasions (Wellings, 2007; Ali et al., 2014a; Hovmøller et al., 2016; Walter et al., 2016) possibly due to a combination of long distance migration capacity (Zadoks, 1961; Brown and Hovmøller, 2002), high rates of mutation from avirulence to virulence (Hovmøller and Justesen, 2007b), adaptation to different climatic conditions (Markell and Milus, 2008; Milus et al., 2009), existence of recombinant and highly diverse populations (Ali et al., 2014a; Thach et al., 2016a) and the potential creation of new variants through a sexual cycle (Jin et al., 2010; Rodriguez-Algaba et al., 2014).

**Figure 1:**
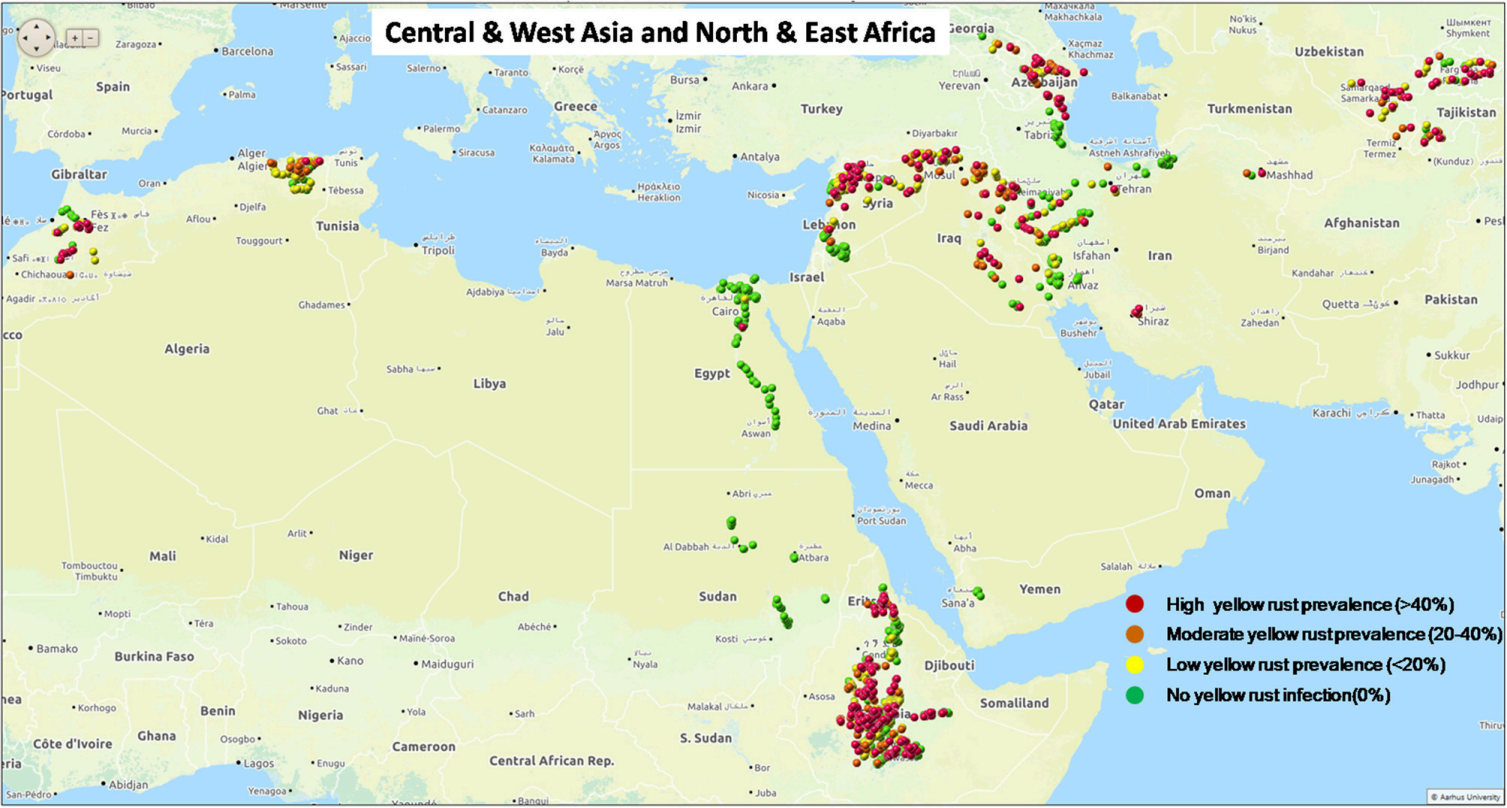
Status of yellow rust prevalence in Central and West Asia and East and North Africa in the 2010 epidemics season.

Yellow rust is a widespread disease across major wheat growing regions with diverse cropping systems, growing seasons and germplasm characteristics (Stubbs, 1985; Manners, 1988; Singh et al., 2004; Wellings, 2011). Resulting losses have been estimated to be at least 5.5 million tons per year at worldwide level (Beddow et al., 2015). Over the last decade a series of regional outbreaks of yellow rust epidemics have been reported worldwide, including Central and West Asia and East and North Africa (Figure 1, Figure S1; www.globalrust.org). A high disease pressure was observed in 2009 and onward in North Africa, particularly in Morocco (Ezzahiri et al., 2009). Since 2010, yellow rust was widely spread in East Africa causing economic losses in low-input farming system (Singh et al., 2016). Widespread epidemics were observed in Tajikistan in 2010 and later on in Uzbekistan and other countries of Central Asia (Rahmatov et al., 2012). In 2010, a high disease prevalence was observed in 2010 in Syria and Lebanon imparting economic losses (El Amil, 2015). These regular epidemics caused not only economic losses and additional need for fungicide sprays, but also threatened seed availability for the next cropping season (Shean, 2010). In Europe, the established *P. striiformis* population has largely been replaced since 2011 by distinct new lineages, generally known as Warrior, Kranich, and Warrior(−), causing increased epidemics on multiple wheat varieties (Rahmatov, 2016), and another lineage associated with epidemics on triticale in 2009–2010, particularly in Scandinavia (Hovmøller et al., 2011). A better understanding of pathogen virulence structure and the divergence of race(s) associated with major epidemic sites at different continents will be useful to facilitate breeding of resistant or less susceptible crop varieties and the development of appropriate disease management strategies based on host resistance (Johnson, 1992; Hawkesford et al., 2013).

New efforts have been made to investigate the yellow rust population genetic structure at worldwide scale, describing the worldwide population subdivisions, sources of invasions and the existence of center of diversity in the Himalayan and near-Himalayan region (Ali et al., 2014a,b; Thach et al., 2016a; Walter et al., 2016). However, the virulence structure of the pathogen has often only been described at country or regional scales, (e.g., Chen, 2005; Zeng and Luo, 2006; Hovmøller and Justesen, 2007a; Wellings, 2007; Bahri et al., 2009b, 2011; Ali et al., 2014c; Hovmøller et al., 2016). Since the work of R.W. Stubbs from 1950s to 1980s (Stubbs, 1988; Thach et al., 2015), only a single study about yellow rust virulence at the international scale has been published (Sharma-Poudyal et al., 2013) and attempts to link virulence and race structure with the recent regional yellow rust epidemic outbreaks in different parts of the world are missing.

The present study allowed an assessment of virulence diversity of *P. striiformis* at the worldwide level and inferring divergence of races prevalent in the recent worldwide yellow rust epidemics. The study was designed to: (i) determine the virulence profile of worldwide collection of *P. striiformis* isolates representing diverse geographical and evolutionary origin (ii) identify predominant races from recent epidemics worldwide and their divergence and prevalence in different geographical regions, and (iii) describe virulence and race diversity at continental scales using representative samples collected worldwide between 2009 and 2015.

## Materials and methods

### Processing of incoming samples at the global rust reference centre

Field samples of yellow rust infected leaves from countries representing five continents were submitted to the Global Rust Reference Centre (GRRC), Aarhus University, Denmark (Table 1). The samples from outside Europe were mostly sent by collaborators working in wheat breeding, rust pathology or agriculture extension generally within the network of the Borlaug Global Rust Initiative whereas European samples were part of ongoing survey activities in Scandinavia and bilateral agreements with GRRC (Table S1). The field samples contained information on date of sampling, location, crop type (winter/spring wheat, barley, triticale etc.) and where possible the host variety and GPS coordinates. The number of samples varied across years and locations partly associated with the epidemic situation and sampling effort in a particular region. A total of 887 samples were virulence phenotyped, representing 35 countries during the period of 2009 to 2015.

**Table 1:** Worldwide *P. striiformis* isolates used to analyze virulence diversity and race divergence during 2009-2015.

**Geographical origin**	**Country**	**No. of isolates**	**Year of sampling**
Europe	Belgium	8	2014
	Bulgaria	4	2015
	Czech Republic	6	2014, 2015
	Denmark	231	2009, 2010, 2011, 2012, 2013, 2014, 2015
	Latvia	2	2015
	Norway	19	2015
	Portugal	3	2013
	Slovakia	4	2014
	Spain	29	2012, 2013, 2014, 2015
	Sweden	156	2009, 2010, 2011, 2012, 2013, 2014, 2015
North America	USA	8	2012, 2013, 2014, 2015
South America	Argentina	5	2010, 2015
	Brazil	5	2010
	Chile	8	2010
	Uruguay	10	2010
North Africa	Algeria	4	2014
	Egypt	5	2013
	Morocco	28	2009, 2013
West Asia	Azerbaijan	19	2009, 2010, 2012, 2015
	Iran	7	2011, 2013, 2014
	Iraq	20	2010, 2011, 2013, 2014
	Lebanon	2	2012
	Syria	28	2009, 2010
	Turkey	5	2011
Central Asia	Tajikistan	17	2010, 2011, 2013
	Uzbekistan	39	2010, 2011, 2013
East Africa	Eritrea	5	2011, 2012
	Ethiopia	50	2010, 2012, 2013, 2014, 2015
	Kenya	47	2009, 2011, 2013, 2014
	Rwanda	27	2014, 2015
	Tanzania	8	2013, 2015
South Asia	Afghanistan	44	2009, 2010, 2011, 2012, 2013, 2014, 2015
	Bhutan	23	2012, 2013, 2015
	Nepal	4	2014, 2015
	Pakistan	6	2014
Total		887	2009, 2010, 2011, 2012, 2013, 2014, 2015

### Isolate revival and multiplication

Field samples were processed for recovery and multiplication using standard GRRC procedures: Infected leaves were kept on moist filter papers in petri dishes under humid conditions at 13°C for 1–2 days to promote formation and release of urediniospores, and then inoculated on susceptible seedlings of wheat lines Cartago, Anja and/or Morocco by rubbing the infected leaf segment on the abaxial side of the leaves. The inoculated seedlings were misted with water and incubated in darkness at 10°C for 24 h under high relative humidity. After incubation the inoculated plants were transferred to quarantine spore-proof greenhouse cabins with a temperature regime of 17°C day and 12°C night and a light regime of 16 h photoperiod of natural light and supplemental sodium light (100 μmol s^−1^ m^−2^) and 8 h dark. The plants were covered with cellophane bags before sporulation to avoid cross contamination. The spores were collected 15–20 days after inoculation and kept in a desiccator for at least 3 days. The urediniospores were then transferred to liquid Nitrogen storage (−196°C) for further virulence phenotyping.

Recovery rates varied according to sampling conditions, treatment after sampling and time between sampling and arrival at GRRC. On average, 53% of samples were successfully recovered. Attempts were made to ensure multiplication of single-genotype samples, and on average only 3–5% of samples showed indication of multiple races, which was detected at time of assessment of the virulence phenotype. In case of multiple/contrasting infection types on individual differential lines, the isolate was sub-cultured based on single lesions, multiplied and re-tested for virulence phenotype confirmation (Thach et al., 2015; Hovmøller et al., 2017).

### Virulence phenotyping

Virulence phenotyping was made on a set of up to 37 wheat yellow rust differential lines, which for most isolates enabled detection of virulence (*v*) corresponding to 19 resistance genes, i.e., *Yr1, Yr2, Yr3, Yr4, Yr5, Yr6, Yr7, Yr8, Yr9, Yr10, Yr15, Yr17, Yr24, Yr25, Yr27, Yr32*, and the resistance specificities in Spaldings Prolific (Sp), Avocet S (AvS), and Ambition (Amb). The differential lines consisted of subsets of the European and World differential sets, near isogenic lines in an Avocet background and additional supplementary European wheat varieties (Johnson et al., 1972; Wellings et al., 2004; de Vallavieille-Pope et al., 2012; Hovmøller et al., 2016).

Virulence phenotyping was made through inoculation of differential lines following previously described protocols (Thach et al., 2015; Hovmøller et al., 2016). Both first and second seedling leaf were considered for virulence phenotyping, where infection type 7–9 on a 0–9 scale (McNeal et al., 1971) were generally considered to reflect compatibility (virulence) and 0–6 incompatibility (avirulence). Conclusions about the phenotype for virulence and avirulence corresponding to the individual resistance genes were in most cases deduced from the infection types on two to three differential lines carrying the considered *Yr*-gene. The virulence profile/phenotype was inferred based on the overall virulence combination, and each distinct virulence phenotype was considered a distinct race.

### Naming system for races associated with epidemics

Prevalent races associated with epidemic outbreaks were assigned a name according to their genetic lineage identified through their molecular genotyping in comparison with the worldwide defined genetic groups (Ali et al., 2014a; Hovmøller et al., 2016; Thach et al., 2016a). A subset of 373 isolates representing epidemics causing races from different geographical regions (Table S2) were microsatellite genotyped (Walter et al., 2016), while a subset of 273 isolates were genotyped with SCAR marker (Hovmøller et al., 2008; Walter et al., 2016). Each lineage, consisting of one or more closely related multi-locus genotypes (“strains”) of a particular race (virulence phenotype), was named *Pst* followed by a forthcoming digit. Virulence variants were designated by the additional virulence observed or (−) in case a new variant had fewer virulences than the first defined race within the considered lineage. *PstS1* and *PstS2* represented two closely related lineages previously defined by AFLP, microsatellite and SCAR markers (Hovmøller et al., 2008; Ali et al., 2014a; Walter et al., 2016). *PstS3* represented a clonal lineage prevalent in southern Europe, North Africa and West Asia (Ali et al., 2014a). Another lineage consisting of races prevalent on triticale in Northern Europe was named *PstS4* (Hovmøller et al., 2008, 2016). *PstS5* consisted of two races within a separate lineage with a specific microsatellite profile as compared to the previously described lineages (Ali et al., 2014a; Hovmøller et al., 2016; Thach et al., 2016a,b). *PstS6* represented a lineage prevalent in East Africa with specific microsatellite profile compared to other worldwide populations (Ali et al., 2014a; Hovmøller et al., 2016; Thach et al., 2016a,b). Recently emerged European lineages, known by the farming community in Europe as Warrior and Kranich (Hovmøller et al., 2016) were named *PstS7* and *PstS8*. Another central Asian lineage related to *PstS5*, which has been associated with epidemics in Central Asia since 2013 was named *Pst*S9. Finally, *Pst*S10 represented a lineage previously known as Warrior(-), first detected in Europe in 2012 and prevalent in most parts of Europe since 2014 (www.wheatrust.org). Many previously detected races in the NW-European population since the late 1950s were part of a single clonal lineage termed *PstS0* (Ali et al., 2014a; Hovmøller et al., 2016; Thach et al., 2016a,b).

### Compilation, analyses and interpretation of data

The virulence phenotype for each isolate was compiled into Excel sheets along with sampling information like country, location, host variety, crop type collection date etc. The compiled data were uploaded to the Wheat Rust Toolbox database (http://wheatrust.org/wheat-rust-toolbox/) for further data archiving, management and display, which was developed under the framework of the Borlaug Global Rust Initiative (www.globalrust.org). Final outputs of the toolbox are freely accessible via the Global Rust Reference Centre (www.wheatrust.org) and the Global Rust Monitoring System (http://rusttracker.cimmyt.org; Hodson et al., 2012; Hansen and Lassen, 2013).

The virulence corresponding to individual resistance genes and the combined virulence phenotype of the race were considered for analyses and interpretation. Diversity in terms of virulence and race was estimated across geographical locations using Simpson diversity index, 1-D (Simpson, 1949), where each individual virulence or each race was considered a different variant at a given geographical region for calculation of virulence and race diversity, respectively. The distribution of virulences and races were assessed across geographical regions and countries and associated with reported epidemic events based on information obtained through the BGRI rust tracker (http://rusttracker.cimmyt.org/) (Hodson et al., 2012; Hansen and Lassen, 2013).

## Results

The virulence phenotyping of 887 *P. striiformis* isolates from 35 countries representing eight geographical regions resulted in the detection of a total of 79 races (virulence phenotypes) from different genetic lineages. The prevalence of races varied widely across regions and none was detected in all regions.

### Divergence in worldwide emerging *P. striiformis* races

**Figure 2:**
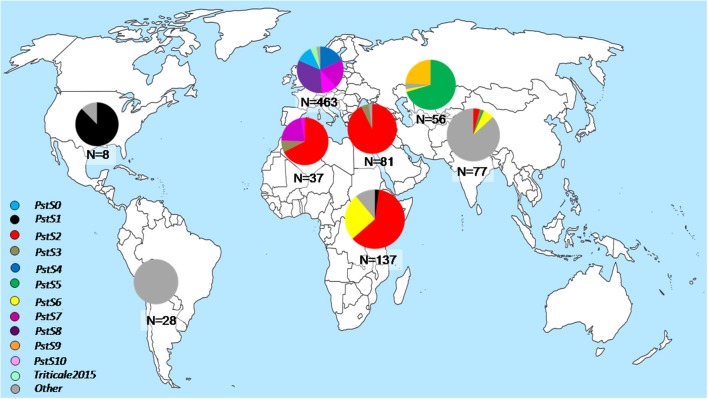
Relative distribution of recently emerged *P. striiformis* races from divergent lineages across eight worldwide geographical regions during the period 2009–2015.

*P. striiformis* remained significantly important around the world during 2009–2015 due to the emergence of races from divergent genetic lineages causing economically important epidemics in various parts of the world (Figure 2). These races represent emerging lineages which have recently been reported as new lineages and are becoming increasingly important epidemic drivers across large geographical area. The previously characterized *PstS1* and *PstS2* and their variants were prevalent at epidemics sites in North America (only *PstS1*), North Africa and West Asia (only *PstS2*) and East Africa (predominantly *PstS2* with detection of *PstS1*), particularly during the epidemics in Morocco in 2009 and Syria in 2009–2010. Three races from distinct lineages, *PstS7, PstS8*, and *PstS10*, were prevalent in Europe since 2011, together covering more than 80% of the investigated isolates. Races from the *PstS3* were detected in Europe, North Africa, West Asia and South Asia. *PstS4* was highly prevalent in epidemics on triticale in 2009–2011 in Europe, particularly in Scandinavia. The Central Asian population was dominated (more than 90%) by *PstS5* and *PstS9*. Races from the related *PstS5* and *PstS9* lineages were highly prevalent in epidemic areas in Central Asia, particularly in the 2010 epidemics in Tajikistan and in later years in Uzbekistan. In addition to *PstS2* variants, the East African population was dominated by a race from the *PstS6* (up to 25%), which was associated with huge epidemics in Ethiopia in 2010 (Table 2). *PstS5* and *PstS6* were also detected in South Asia, but in very low frequencies. Races from these divergent lineages had characteristic virulence phenotypes along with their microsatellite genotype, which was clearly divergent from one another and from other races (Table 2, Table S3). The race of *PstS7* (a.k.a. Warrior) was the most divergent from other lineages based on the virulence phenotype, while the race of *PstS8* (a.k.a. Kranich) was related with races of *PstS5* prevalent in Central Asia. The two closely related and widely prevalent lineages *PstS1* and *PstS2* were the most diversified lineages with multiple variants often differing with a single or few virulences.

**Table 2:** Characteristics of regionally important *P. striiformis* lineages and virulence variants prevalent during 2009–2015.

**Genetic lineage**	**Lineage variant**	**Virulence phenotype**	**Prevalence across geographical regions**
PstS0^*^	Robigus	1,2,3,4,-,-,-,-,9,-,-,17,-,25,-,32,-,AvS,-	Europe
	Solstice/Oakley	1,2,3,4,-,6,-,-,9,-,-,17,-,25,-,32,-,AvS,-	Europe
	Tulsa	-,-,3,4,-,6,-,-,-,-,-,-,-,25,-,32,-,AvS,-	Europe
	Brigadier,v4	1,2,3,4,-,-,-,-,9,-,-,17,-,25,-,-,-,AvS,-	Europe
PstS1^**^	*PstS1,v10,v24,v27*	-,2,-,-,-,6,7,8,9,10,-,-,24,25,27,-,-,AvS,-	East Africa
	*PstS1,v17*	-,2,-,-,-,6,7,8,9,-,-,17,-,25,-,-,-,AvS,-	North America
	*PstS1,v17,v27*	-,2,-,-,-,6,7,8,9,-,-,17,-,25,27,-,-,AvS,-	North America
	*PstS1,v3,v17,v27*	-,2,3,-,-,6,7,8,9,-,-,17,-,25,27,-,-,AvS,-	North America
	*PstS1,v3,v17,v27,v32*	-,2,3,-,-,6,7,8,9,-,-,17,-,25,27,32,-,AvS,-	North America
*PstS2*	*PstS2*	-,2,-,-,-,6,7,8,9,-,-,-,-,25,-,-,-,AvS,-	East Africa, West Asia, South Asia
	*PstS2,v1*	1,2,-,-,-,6,7,8,9,-,-,-,-,25,-,-,-,AvS,-	East Africa, West Asia
	*PstS2,v3*	-,2,3,-,-,6,7,8,9,-,-,-,-,25,-,-,-,AvS,-	East Africa
	*PstS2,v27*	-,2,-,-,-,6,7,8,9,-,-,-,-,25,27,-,-,AvS,-	East Africa, West Asia, North Africa
	*Pst2,v1,v27*	1,2,-,-,-,6,7,8,9,-,-,-,-,25,27,-,-,AvS,-	East Africa, West Asia
	*PstS2,v3,v27*	-,2,3,-,-,6,7,8,9,-,-,-,-,25,27,-,-,AvS,-	East Africa
	*PstS2,v10,v24*	-,2,-,-,-,6,7,8,9,10,-,-,24,25,-,-,-,AvS,-	East Africa, West Asia
	*PstS2,v3,v10,v24,v27*	-,2,3,-,-,6,7,8,9,10,-,-,24,25,27,-,-,AvS,-	East Africa
	*PstS2,v10,v24,v27*	-,2,-,-,-,6,7,8,9,10,-,-,24,25,27,-,-,AvS,-	West Asia
*PstS3*	*PstS3*	-,-,-,-,-,6,7,8,-,-,-,-,-,-,-,-,-,AvS,-	North Africa, West Asia
	*PstS3,v10,v24*	-,-,-,-,-,6,7,8,-,10,-,-,24,-,-,-,-,AvS,-	West Asia
	*PstS3(-)*	-,-,-,-,-,6,7,8,-,-,-,-,-,-,-,-,-,-,-	Europe, South Asia
PstS4^***^	*PstS4*	-,2,-,-,-,6,7,8,-,10,-,-,24,-,-,-,-,-,-	Europe
*PstS5*	*PstS5*	1,2,3,4,-,6,-,-,9,-,-,-,-,25,-,32,-,AvS,Amb	Central Asia
	*PstS5,v17*	1,2,3,4,-,6,-,-,9,-,-,17,-,25,-,32,-,AvS,Amb	Central Asia, South Asia
*PstS6*	*PstS6*	1,2,-,-,-,6,7,-,9,-,-,17,-,-,27,-,-,AvS,-	East Africa, Central Asia, South Asia
*PstS7*	Warrior	1,2,3,4,-,6,7,-,9,-,-,17,-,25,-,32,Sp,AvS,Amb	Europe, North Africa
*PstS8*	Kranich	1,2,3,-,-,6,7,8,9,-,-,17,-,25,-,32,-,AvS,Amb	Europe
*PstS9*	*PstS9*	1,2,3,4,-,6,-,-,9,-,-,-,-,25,27,32,-,AvS,Amb	Central Asia, South Asia
	*PstS9,v17*	1,2,3,4,-,6,-,-,9,-,-,17,-,25,27,32,-,AvS,Amb	Central Asia
*PstS10*	Warrior(−)	1,2,3,4,-,6,7,-,9,-,-,17,-,25,-,32,Sp,AvS,-	Europe, North Africa

* *Represents the clonal NW European lineage refering to many additional races prevalent in Europe up to 2010*.

** *Reference isolates from PstS1 provided by Gene Milus and extensively described in Milus et al. (2015a,b)*.

*** *Synonymous to “Triticale Aggressive” in Hovmøller et al. (2016); v24/Avr24 could not be assessed due to avirulence on Avocet S*.

### Diversity in virulence and races across geographical regions worldwide

Out of the 19 *Yr* genes investigated, virulence were observed to 17 when the overall worldwide population was considered (Table 3, Table S4). Virulence to *Yr5* and *Yr15* were absent, while virulence to *Yr10* and *Yr24* was rare (up to 12%) in most areas except for samples from Triticale, e.g., *PstS4*. None of the virulences was fixed in the overall worldwide population, however, virulence to *Yr2, Yr6, Yr7, Yr9*, and *Yr25* were generally in high frequencies and across many regions (>70%). Virulence frequencies varied substantially across regions. In addition to *Yr5* and *Yr15* in all regions, virulence was not detected to *Yr27* in Europe; to *Yr3, Yr4, Yr17, Yr32, YrSp*, and *YrAmb* in West Asia; *Yr10* and *Yr24* in North Africa and South Asia; to *Yr10, Yr24*, and *YrSp* in Central Asia; and to *Yr10, Yr32*, and *YrSp* in East Africa.

**Table 3:** Diversity for race and virulence in *P. striiformis* populations sampled from worldwide geographical regions during 2009–2015.

**Geographical region**	**No. of isolates tested**	**No. of distinct races**	**No. of virulences detected**	**Prevalence of most abundant race**	Race diversity^*^	**Yr genes not defeated in isolates investigated**
Europe	463	17	16	152	0.799	*Yr5, Yr15, Yr27*
South America	28	3	11	17	0.538	*Yr5, Yr8, Yr10, Yr15, Yr24, Yr32, YrSp, YrAmb*
North Africa	37	4	15	25	0.489	*Yr5, Yr10, Yr15, Yr24*
West Asia	81	9	11	46	0.592	*Yr3, Yr4, Yr5, Yr15, Yr17, Yr32, YrSp, YrAmb*
Central Asia	56	7	14	28	0.659	*Yr5, Yr10, Yr15, Yr24, YrSp*
East Africa	137	22	14	42	0.822	*Yr4, Yr5, Yr15, Yr32, YrSp*
South Asia	77	31	15	10	0.941	*Yr5, Yr10, Yr15, Yr24*
Overall population	887	79	17	152	0.927	*Yr5, Yr15*

* *Based on Simpson diversity index “1-D” (Simpson, 1949)*.

Geographical regions varied with respect to diversity, both in terms of virulences and races detected (Table 3, Table S4). The maximum number of virulences, regardless of combination in single races, was detected in Europe (16), while the minimum was observed in West Asia and South America (11). The highest number of races was detected in the pathogen center of diversity in South Asia (31) and the minimum in South America (3). Similarly, race diversity was the highest in South Asia (0.941) and lowest in North Africa (0.489). Europe, with the highest sampling intensity, had a high race diversity (0.799) due to the presence of both pre-2011 NW European races and post-2011 races like *PstS7, PstS8*, and *PstS10*.

### Races prevalent across worldwide geographical regions

The 79 races detected in the worldwide population represented a wide range of races from the simplest (avirulent on all the tested *Yr* genes) to complex races like the one in *PstS7* and *PstS8* lineages (Tables 4A–**D**). None of the races was present on all continents representing eight geographical regions. While races from the emerging lineages like *PstS2, PstS4, PstS5, PstS6, PstS7, PstS8, PstS9*, and *PstS10*, were prevalent in distinct epidemic situations, most of the other races (34 out of 79) were detected only once (Table 4, Table S5).

**Table 4A:** Prevalence of *P. striiformis* races detected in Europe during 2009–2015.

**Virulence phenotype**	**Lineage variant**	Prevalence in Europe^**^	**Detection in other geographical populations**
1,2,3,-,-,6,7,8,9,-,-,17,-,25,-,32,-,AvS,Amb	*PstS8*	152	
1,2,3,4,-,6,7,-,9,-,-,17,-,25,-,32,Sp,AvS,Amb	*PstS7*	94	North Africa (8)
-,2,-,-,-,6,7,8,-,10,-,-,^*^,-,-,-,-,-,-	*PstS4*	84	
1,2,3,4,-,6,7,-,9,-,-,17,-,25,-,32,Sp,AvS,-	*PstS10*	48	Europe (1)
-,-,3,4,-,6,-,-,-,-,-,-,-,25,-,32,-,AvS,-	Tulsa	32	
-,2,-,-,-,6,7,8,9,-,-,-,-,-,-,-,-,AvS,-	Triticale-2015	20	
1,2,3,4,-,6,-,-,9,-,-,17,-,25,-,32,-,AvS,-	Solstice/Oakley	14	
1,2,3,4,-,-,-,-,9,-,-,17,-,25,-,32,-,AvS,-	Robigus	6	
-,-,-,-,-,-,-,-,-,-,-,-,-,-,-,-,-,-,-	Non-wheat	4	
1,2,3,4,-,-,-,-,9,-,-,17,-,25,-,-,-,AvS,-	Brigadier,v4	1	
-,-,-,-,-,6,7,8,-,-,-,-,^*^,-,-,-,-,-,-	*PstS3(−)*	1	South Asia (1)
-,2,3,-,-,6,7,8,-,-,-,-,-,25,-,32,-,AvS,-	–	2	
-,2,-,-,-,6,7,8,-,-,-,-,-,25,-,-,-,AvS,-	–	1	East Africa (1)
1,2,3,-,-,-,-,-,9,-,-,17,-,25,-,-,-,AvS,-	–	1	
-,2,-,-,-,6,7,8,-,-,-,-,-,25,-,32,-,AvS,-	–	1	
1,-,3,4,-,6,-,-,-,-,-,-,-,25,-,32,-,AvS,-	–	1	
1,2,3,-,-,6,-,-,9,-,-,17,-,25,-,32,-,AvS,-	–	1	

* *v24/Avr24 could not be assessed due to avirulence to Avocet S*.

** *High sampling activity in Scandinavia resulted in higher than expected frequency of PstS8 (Kranich) in Europe*.

*Figures and symbols designate virulence and avirulence (–) corresponding to yellow rust resistance genes: Yr1, Yr2, Yr3, Yr4, Yr5, Yr6, Yr7, Yr8, Yr9, Yr10, Yr15, Yr17, Yr24, Yr25, Yr27, Yr32, and the resistance specificity of Spalding Prolific (Sp), Avocet S (AvS), and Ambition (Amb), respectively*.

A total of 17 races were detected in the 462 isolates representing the European population during 2009–2015. *PstS8, PstS7, PstS10*, and *PstS4* were the most frequent representing more than 80 percent of the population, while the pre-2011 races remained in low prevalence during the period (Figure 2, Table 4A). Among these 17 races, seven were detected only once in Europe during the period. Four, including *PstS7* and *PstS8*, were re-sampled in other geographical regions.

In South America, three races were detected among the 28 tested isolates, none of which was detected in any other geographical region (Table 4B). Three *PstS1* races were present in the isolates from North America, along with another race, none of which were re-sampled in any other geographical region (Table 4B).

**Table 4B:** Prevalence of *P. striiformis* races detected in North America and South America during 2009–2015.

**Virulence phenotype**	**Lineage variant**	**North America**	**South America**
-,2,-,-,-,6,7,8,9,-,-,17,-,25,-,-,-,AvS,-	*PstS1,v17*	2	–
-,2,-,-,-,6,7,8,9,-,-,17,-,25,27,-,-,AvS,-	*PstS1,v17,v27*	2	–
-,2,3,-,-,6,7,8,9,-,-,17,-,25,27,32,-,AvS,-	*PstS1,v3,v17,v27,v32*	2	–
-,2,3,-,-,6,7,8,9,-,-,17,-,25,27,-,-,AvS,-	*PstS1,v3,v17,v27*	1	–
1,2,3,-,-,6,7,8,9,-,-,17,-,25,27,-,-,AvS,Amb	–	1	–
-,2,3,4,-,6,7,-,-,-,-,-,-,25,-,-,-,AvS,-	–	–	17
1,2,3,4,-,6,7,-,9,-,-,17,-,25,27,-,-,AvS,-	–	–	8
1,-,-,-,-,-,-,-,-,-,-,-,^*^,-,-,-,-,-,-	–	–	3

* *v24/Avr24 could not be assessed due to avirulence to Avocet S*.

*Figures and symbols designate virulence and avirulence(–) corresponding to yellow rust resistance genes: Yr1, Yr2, Yr3, Yr4, Yr5, Yr6, Yr7, Yr8,Yr9, Yr10, Yr15, Yr17, Yr24, Yr25, Yr27, Yr32, and the resistance specificity of Spalding Prolific (Sp), Avocet S (AvS), and Ambition (Amb), respectively*.

A total of 33 races were detected in the larger area comprising Central Asia, West Asia, North Africa, and East Africa; seven of these were shared by most of these regions (Table 4C). North Africa and West Asia was dominated by races from the *PstS2* lineage. In East Africa, *PstS2* and *PstS6* were the most prevalent during the studied period. Six races were typical to Central Asia and 18 were typical to East Africa, with limited re-sampling in other geographical regions. These regions had diverse races ranging from virulence to a single *Yr* gene (e.g., *v27*) to races of diverse virulence phenotypes (e.g., *PstS9,v17*).

**Table 4C:** Prevalence of *P. striiformis* races detected in Central Asia, West Asia and East and North Africa during 2009–2015.

Virulence phenotype^*^	**Lineage variant**	**Prevalence in different geographical regions**				**Detection in other geographical populations**
		**North Africa**	**West Asia**	**Central Asia**	**East Africa**	
-,2,-,-,-,6,7,8,9,-,-,-,-,25,27,-,-,AvS,-	*PstS2,v27*	25	46	–	9	
1,2,3,4,-,6,7,-,9,-,-,17,-,25,-,32,Sp,AvS,Amb	*PstS7*	8	–	–	–	Europe (93)
-,-,-,-,-,6,7,8,-,-,-,-,-,-,-,-,-,AvS,-	*PstS3*	3	3	–	–	
1,2,3,4,-,6,7,-,9,-,-,17,-,25,-,32,Sp,AvS,-	*PstS10*	1	–	–	–	Europe (48)
-,2,-,-,-,6,7,8,9,-,-,-,-,25,-,-,-,AvS,-	*PstS2*	–	23	–	9	South Asia (3)
-,-,-,-,-,6,7,8,-,10,-,-,24,-,-,-,-,AvS,-	*PstS3,v10,v24*	–	1	–	–	
-,-,-,-,-,6,7,8,-,10,-,-,24,-,27,-,-,AvS,-	–	–	1	–	–	
1,2,-,-,-,6,7,-,-,-,-,-,-,-,-,-,-,AvS,-	–	–	1	–	–	
1,2,3,4,-,6,-,-,9,-,-,-,-,25,-,32,-,AvS,Amb	*PstS5*	–	–	28	–	
1,2,3,4,-,6,-,-,9,-,-,-,-,25,27,32,-,AvS,Amb	*PstS9*	–	–	9	–	South Asia (1)
1,2,3,4,-,6,-,-,9,-,-,17,-,25,27,32,-,AvS,Amb	*PstS9,v17*	–	–	14	–	
1,2,3,4,-,6,-,-,9,-,-,17,-,25,-,32,-,AvS,Amb	*PstS5,v17*	–	–	2	–	South Asia (1)
1,2,-,-,-,6,7,8,-,-,-,-,-,-,27,-,-,AvS,-	–	–	–	1	–	
1,2,-,-,-,6,7,8,-,-,-,-,-,25,27,-,-,AvS,-	–	–	–	1	–	
1,2,-,-,-,6,7,8,9,-,-,-,-,25,27,-,-,AvS,-	*Pst2,v1,v27*	–	1		42	
1,2,-,-,-,6,7,-,9,-,-,17,-,-,27,-,-,AvS,-	*PstS6*	–	–	1	35	South Asia (5)
-,2,3,-,-,6,7,8,9,-,-,-,-,25,-,-,-,AvS,-	*PstS2,v3*	–	–	–	9	
-,2,-,-,-,6,7,8,9,10,-,-,24,25,27,-,-,AvS,-	*PstS2,v10,v24,v27*	–	–	–	4	
-,2,-,-,-,6,7,8,9,10,-,-,24,25,-,-,-,AvS,-	*PstS2,v10,v24*	–	4	–	4	
1,2,-,-,-,6,7,8,9,-,-,-,-,25,-,-,-,AvS,-	*PstS2,v1*	–	1	–	3	
-,2,3,-,-,6,7,8,-,-,-,-,-,25,27,-,-,AvS,-	–	–	–	–	1	
-,2,3,-,-,6,7,8,9,-,-,-,-,25,27,-,-,AvS,-	*PstS2,v3,v27*	–	–	–	8	
-,2,-,-,-,6,7,8,-,10,-,-,24,-,27,-,-,AvS,-	–	–	–	–	3	
-,2,-,-,-,6,7,8,-,-,-,-,-,25,27,-,-,AvS,-	–	–	–	–	2	
-,-,-,-,-,-,-,-,-,-,-,-,-,-,27,-,-,-,-	–	–	–	–	1	
-,-,-,-,-,6,7,8,9,10,-,-,24,-,-,-,-,-,-	–	–	–	–	1	
-,2,-,-,-,6,7,8,-,-,-,17,-,-,-,-,-,AvS,-	–	–	–	–	1	
-,2,-,-,-,6,7,8,-,10,-,-,-,-,-,-,-,-,-	–	–	–	–	1	
-,2,3,-,-,6,7,8,-,10,-,-,24,25,27,-,-,AvS,-	–	–	–	–	1	
1,2,-,-,-,6,7,-,-,-,-,17,-,25,-,-,-,AvS,-	–	–	–	–	1	
1,2,-,-,-,6,7,8,-,-,-,17,-,25,-,-,-,AvS,-	–	–	–	–	1	
-,2,-,-,-,6,7,8,-,-,-,-,-,25,-,-,-,AvS,-	–	–	–	–	1	Europe (1)

* *v24/Avr24 could not be assessed due to avirulence to Avocet S*.

*Figures and symbols designate virulence and avirulence(–) corresponding to yellow rust resistance genes: Yr1, Yr2, Yr3, Yr4, Yr5, Yr6, Yr7, Yr8, Yr9, Yr10, Yr15, Yr17, Yr24, Yr25, Yr27, Yr32, and the resistance specificity of Spalding Prolific (Sp), Avocet S (AvS), and Ambition (Amb), respectively*.

A total of 31 races were detected in South Asia, varying from very simple races with two virulences (e.g., *v8*,*AvS*) to complex races with virulence to 13 *Yr* genes (e.g., *v1,2,3,4,6,7,9,17,25,32,Sp,AvS,Amb*). None of the races dominated the South Asian population. Five of the South Asian races were re-sampled in other geographical regions and 18 were detected only once in South Asia (Table 4D).

**Table 4D:** Prevalence of *P. striiformis* races detected in South Asia during 2009–2015.

**Virulence phenotype**	**Lineage variant**	**Prevalence in South Asia**	**Detection in other geographical populations**
1,2,-,-,-,6,7,-,9,-,-,17,-,-,27,-,-,AvS,-	*PstS6*	5	Central Asia (1), East Africa (35)
-,2,-,-,-,6,7,8,9,-,-,-,-,25,-,-,-,AvS,-	*PstS2*	3	West Asia (23), East Africa (9)
1,2,3,4,-,6,-,-,9,-,-,17,-,25,-,32,-,AvS,Amb	*PstS5,v17*	1	Central Asia (2)
1,2,3,4,-,6,-,-,9,-,-,-,-,25,27,32,-,AvS,Amb	*PstS9*	1	Central Asia (9)
-,-,-,-,-,6,7,8,-,-,-,-,^*^,-,-,-,-,-,-	*PstS3(−)*	1	Europe(1)
-,2,-,4,-,6,7,8,-,-,-,17,-,-,27,32,-,AvS,-	–	10	
1,2,3,4,-,6,7,-,9,-,-,17,-,25,-,32,Sp,AvS,Amb^**^	–	8	
1,2,-,-,-,6,7,8,-,-,-,17,-,-,-,-,-,AvS,-	–	6	
1,-,-,-,-,6,7,-,9,-,-,-,-,-,27,-,-,AvS,-	–	5	
1,2,-,-,-,6,7,8,9,-,-,17,-,-,27,-,-,AvS,-	–	4	
1,2,-,4,-,6,7,8,9,-,-,17,-,-,27,-,-,AvS,-	–	4	
-,2,3,-,-,6,7,8,9,-,-,-,-,25,27,-,-,AvS,-	–	3	
-,-,-,-,-,-,-,8,-,-,-,-,-,-,-,-,-,AvS,-	–	3	
1,-,-,-,-,-,7,-,9,-,-,-,-,-,27,-,-,AvS,-	–	3	
1,2,-,4,-,6,7,8,-,-,-,-,-,-,-,-,-,AvS,-	–	3	
1,2,-,4,-,6,7,8,-,-,-,17,-,-,-,-,-,AvS,-	–	2	
1,2,-,-,-,6,7,8,9,-,-,-,-,25,27,-,-,AvS,-	–	1	
-,2,3,-,-,6,7,8,9,-,-,17,-,25,-,-,-,AvS,Amb	–	1	
-,2,-,-,-,6,-,8,-,-,-,-,-,-,-,-,Sp,AvS,-	–	1	
-,2,-,-,-,6,7,-,-,-,-,-,-,25,-,-,-,AvS,-	–	1	
-,2,-,-,-,6,7,-,-,-,-,17,-,25,-,-,-,AvS,Amb	–	1	
-,2,-,4,-,6,7,8,9,-,-,17,-,-,27,-,-,AvS,-	–	1	
-,2,-,4,-,6,7,8,9,-,-,17,-,-,27,32,-,AvS,-	–	1	
1,-,-,-,-,-,7,-,9,-,-,-,-,-,-,-,-,AvS,-	–	1	
1,-,-,-,-,6,7,8,-,-,-,-,-,-,-,-,-,AvS,-	–	1	
1,-,-,-,-,6,7,8,9,-,-,-,-,-,27,-,-,AvS,-	–	1	
1,2,-,-,-,6,7,-,-,-,-,-,-,25,-,-,-,AvS,-	–	1	
1,2,-,4,-,6,7,8,-,-,-,17,-,-,27,-,-,AvS,Amb	–	1	
1,2,-,4,-,6,7,8,9,-,-,17,-,-,27,32,-,AvS,-	–	1	
1,2,3,4,-,6,-,8,9,-,-,-,-,25,-,32,-,AvS,Amb	–	1	
1,2,3,-,-,-,-,-,9,-,-,17,-,25,-,-,-,AvS,-	–	1	

* *v24/Avr24 could not be assessed due to avirulence to Avocet S*.

** *Clearly different genotypic profile from PstS7 lineage*.

*Figures and symbols designate virulence and avirulence(–) corresponding to yellow rust resistance genes: Yr1, Yr2, Yr3, Yr4, Yr5, Yr6, Yr7, Yr8, Yr9, Yr10, Yr15, Yr17, Yr24, Yr25, Yr27, Yr32, and the resistance specificity of Spalding Prolific (Sp), Avocet S (AvS), and Ambition (Amb), respectively*.

## Discussion

We report on the race and virulence structure of *P. striiformis* isolates from five continents including several yellow rust epidemic areas during 2009 to 2015. Our results showed that races from relatively few divergent lineages were associated with huge yellow rust epidemics in different parts of the world, resulting in economic losses in the respective regions. High frequencies of virulence to widely deployed resistance genes in the regions, and the absence of virulences to other host resistance genes, are discussed in the context of sustainable use of host resistance in crop varieties. Finally, the impact of invasions on shaping the pathogen population across geographical regions is discussed. The results were based on a set of samples from important wheat varieties and breeding lines in different geographical regions and years. Comparable sample sizes were considered for most of the regions, except North America where isolates consisted of important references from previous studies (Milus et al., 2015a,b). The study did not include the Australian population, which is known to be dominated by races emerging from an incursion of a particular race from NW-Europe in 1978 (Wellings and McIntosh, 1990) and *PstS1* related races (Wellings, 2007; Hovmøller et al., 2008). Northern Europe was over-represented, due to ongoing, intensive surveillance activities (Hovmøller et al., 2015).

### Divergent lineages associated with regional epidemics worldwide

Our results indicated the emergence of races from few divergent lineages in the recent *P. striiformis* outbreaks during 2009–2015, associated with substantial economic losses in various parts of the world (Beddow et al., 2015). These included races from the regionally prevalent lineages *PstS1, PstS2, PstS4, PstS5, PstS6, PstS7, PstS8*, and *PstS10*. Races from the *PstS2* lineage, which emerged in early 2000s (Hovmøller et al., 2008; Milus et al., 2009; Walter et al., 2016), were responsible for severe epidemics in North Africa e.g., Morocco 2009 (Ezzahiri et al., 2009) and West Asia, e.g., Syria 2010 (El Amil, 2015). Different variants of this lineage have successfully been established in North Africa and West Asia and further acquired virulence to *Yr1, Yr3, Yr10*, and *Yr27*, some of which have been widely deployed (Singh et al., 2004; El Amil, 2015). In 2009, substantial disease epidemics were observed on triticale in Europe, particularly in Scandinavia, due to the race (*v2,6,7,8,10*) from the *PstS4* lineage. In Central Asia, where severe epidemics were observed in Tajikistan in 2010 and later on in Uzbekistan and other countries (Rahmatov, 2016), the race *v1,2,3,4,6, 9,25,32,AvS,Amb* and its variants from the *PstS5* and *PstS9* lineages were frequently observed. Similarly, the race *v1,2,6,7,9,17,27,AvS* from the *PstS6* was considered responsible for the epidemics in East Africa in 2010 and onwards, particularly in Ethiopia. In Europe since 2011, races from distinct lineages *PstS7, PstS8*, and *PstS10*, resulted in an ongoing replacement of the pre-2011 European population, being associated with epidemics in many countries (Sørensen et al., 2014; Hovmøller et al., 2016). All of these regionally important lineages had a characteristic microsatellite genotype (Ali et al., 2014a; Hovmøller et al., 2016; Walter et al., 2016) as well as divergent virulence profiles, often reflecting the resistance genes deployed in the region.

### Worldwide race and virulence diversity

Seventy-nine races were detected in the worldwide *P. striiformis* population, here represented by 887 isolates from 35 countries and eight geographical regions. Although none of the races were detected in all geographical regions, *PstS7* and *PstS2* variants (Hovmøller et al., 2016; Walter et al., 2016) were found in several, distant geographical regions, reflecting the long-distance dispersal capacity of rust pathogens (Zadoks, 1961; Hermansen, 1968; Brown and Hovmøller, 2002; Hovmøller et al., 2008; Ali et al., 2014a). Interestingly, 35 races were detected only once in the worldwide population, and the overall population was dominated by the above described genetic lineages associated with regional epidemics, which resulted in economic losses (Wellings, 2011; Beddow et al., 2015; Singh et al., 2016). In many of the geographical regions, relatively low race diversity was observed along with predominance of regionally important lineages. In East Africa, despite high race diversity, the overall population was dominated by races from the *PstS2* and the *PstS6* lineage, which contained virulence to *Yr17* and *Yr27*, two widely deployed resistance genes in the region (Singh et al., 2004; El Amil, 2015). In the South Asian recombinant population (Ali et al., 2014a; Thach et al., 2016a), a high race diversity was observed with no clear prevalence of any particular race. Recombination in a highly diverse population with temporal maintenance through a sexual cycle may generate new variants, including those carrying virulence to the deployed resistance genes. This will maintain the high virulence and race diversity in a recombinant population as observed in China and South Asia (Mboup et al., 2009; Duan et al., 2010; Ali et al., 2014c), even if the related host resistance is not deployed.

### Worldwide virulence structure in the context of host resistance deployment

The observed worldwide virulence structure could be explained to a large extent by the regional deployment of host resistance (Table S6). Virulence to most of the considered resistance genes was observed in Europe, reflecting the large-scale deployment of these genes in Europe in the past (Bayles et al., 2000; Hovmøller, 2007; de Vallavieille-Pope et al., 2012; Hovmøller et al., 2016). The West and South Asian population showed fixation or a very high frequency of virulence to resistance genes widely deployed in the region like *Yr2, Yr6, Yr7, Yr8, Yr9, Yr25*, and *Yr27* (Singh et al., 2004; Bahri et al., 2011; El Amil, 2015). Interestingly *Yr5* and *Yr15* were the only resistance genes to which virulence was not observed in this study. These two genes have so far very rarely been reported deployed on large scale (Singh et al., 2004; Chen, 2005; Hovmøller, 2007; Bahri et al., 2011; de Vallavieille-Pope et al., 2012; El Amil, 2015). Nonetheless, virulence toward *Yr5* and *Yr15* do exist in the center of diversity as well as in spontaneous virulence mutants elsewhere (Wellings and McIntosh, 1990; Hovmøller, 2007; Ali et al., 2014c), but as yet neither has been subject to strong selection by host resistance. The role of selection is further reflected by the predominance of races from major lineages carrying virulences to deployed resistance genes, even in populations with high race diversity, e.g., races from *PstS6* carrying *v17* and *v27* dominating the East African population. These results emphasize the role of host selection on the virulence structure of pathogen populations.

The strength of host selection may be reduced by the use of additional resistance genes with minor effect along with diversification at landscape level (Brown, 2015). Minor genes resulting in partial and adult plant resistance would delay the epidemic development, but also the pace of evolution of pathogen virulence (Hovmøller et al., 1997; Pinnschmidt, 2014). The accumulation of several minor genes can provide substantial disease control (Singh et al., 2004, 2016) and recent studies have proven that several components of resistance, including those with minor effects played a significant role in protecting European wheat varieties against the races of *PstS2* (Walter et al., 2016), *PstS3* (Mboup et al., 2012), and *PstS7* (Sørensen et al., 2014). Efforts have been done on partial resistance assessment in various breeding materials in different parts of the world (Niks, 1987; Hovmøller, 2007; Pathan and Park, 2007; Ali et al., 2009). However, the components of partial resistance in regionally important varieties are often unknown, particularly in Asia and Africa, where most of the varieties are often developed from introduced materials. Diversification at the field level by use of multilines and variety mixtures may also reduce disease epidemics (Mundt and Browning, 1985; Wolfe, 1985; Zhu et al., 2000) and possibly the rate of increase of new virulence, particularly if virulence is associated with a cost (Hovmøller et al., 1997; McDonald and Linde, 2002; Bahri et al., 2009a; Brown, 2015). Thus, considering information about the virulence diversity in prevailing pathogen populations will enable to combine resistance with major and minor effects along with diversification of their deployment at field and landscape levels.

### Worldwide virulence structure in relation to genetic structure

Our results added into our knowledge on the global landscape of *P. striiformis*. The Himalayan and near Himalayan populations have been shown to be recombinant and the center of diversity (Ali et al., 2014a,b; Thach et al., 2016a), which is endorsed by the high race diversity observed in the region. The Central Asian population has been shown to be closely related in 2000–2005 to the West Asian population based on samples from Kazakhstan, Kyrgyzstan, and Uzbekistan (Hovmøller et al., 2008; Ali et al., 2014a), which however was replaced by races from the new *PstS5* causing widespread epidemics in the region since 2010. Races of *PstS5* and *PstS9* are now widely prevalent in the Central Asian region. West Asia has been reported to be invaded by *PstS2* (Hovmøller et al., 2008; Walter et al., 2016), which has now successfully been established in the region, reducing the overall diversity as observed in the population before 1990 (Thach et al., 2016a). The East African population, which was closely related to the Middle Eastern population (Ali et al., 2014a; Thach et al., 2016a), was in this study dominated by *PstS6*. The Mediterranean population has been reported to represent a sink for different races with overall selective advantages (Enjalbert et al., 2005; Thach et al., 2016a). Mediterranean races prevalent in the region have been reported to be replaced by *PstS2* in post-2000 epidemics (Hovmøller et al., 2008; Bahri et al., 2009b; Ali et al., 2014a). Since the appearance of the *PstS7* (a.k.a. Warrior) in Europe, it has spread to North Africa and has become widely established in the region (Hovmøller et al., 2016). Indeed the European population has been shown to be replaced by the *PstS7* and *PstS8* lineages since 2011, which was confirmed in our study (Hovmøller et al., 2016). Regional studies over several years, based on both molecular and virulence data along with consideration of locally deployed host resistance, would further improve our understanding on the changes in global landscape of *P. striiformis*.

## Conclusion

We report virulence and race diversity of worldwide *P. striiformis* populations, with the emphasis on races from regionally prevalent lineages causing epidemic outbreaks resulting in widespread economic losses for wheat production. Although resistance gene deployment in consideration of pathogen population variability played a significant role in protecting European wheat against the pathogen (Mboup et al., 2012; Sørensen et al., 2014; Walter et al., 2016), the emergence and prevalence of races from few divergent lineages highlights the lack of predictability of invasive races in terms of their origin and adaptability. This underlines the need for collaborative efforts from all stakeholders to understand the biology of crop pathogens, drivers of epidemics, surveillance of the pathogen population and vulnerability of host varieties. Concordance of virulence structure and establishment of certain races with regionally deployed host resistance emphasized the role of host selection on pathogen virulence structure and emphasized the need for greater regional and local diversification of host resistance. Efficient sharing of knowledge, germplasm, rust diagnostic facilities and information at national, regional and continental scales will be crucial to meet future challenges by the yellow rust pathogen.

## Author contributions

SA, KN, DH, and MH contributed to collection of isolates; JR, TT, CS, and MH assisted in recovery and multiplication of isolates and performed the race phenotyping; JR and MH interpreted and quality controlled the race phenotype data; SA, JR, JH, PL, DH, and MH compiled and uploaded data into the database; SA, JH, and MH analyzed the data and designed the research; SA, TT, AJ, and MH identified and defined the lineages by molecular genotyping; SA and MH wrote the manuscript. All authors read and contributed to the revision of the manuscript.

### Conflict of interest statement

The authors declare that the research was conducted in the absence of any commercial or financial relationships that could be construed as a potential conflict of interest.

## Supplementary material

Figure S1 Yellow rust epidemics in across four geographical regions (i.e., central and west Asia and East and North Africa) in the 2010 epidemics season.

Table S1 List of collaborators who contributed to the global collection of *P. striiformis* studied to understand the worldwide pathogen population structure during 2009–2015.

Table S2 Details of isolates of *P. striiformis* virulence phenotyped, SSR genotyped and SCAR genotyped to decipher pathogen's worldwide viulence structure.

Table S3 Molecular genotyping profiles of representative *P. striiformis* isolates of worldwide emerging lineages prevalent during 2009–2015.

Table S4 Percentage (%) of different virulences in worldwide *P. striiformis* population samplescollected during 2009–2015.

Table S5 Worldwide prevalence of *P. striiformis* racesacross eight geographical regions during wheat growing seasons in the period 2009–2015.

Table S6 Overview of Yr resistance genes deployment across different geographical regions worldwide, considering epidemics associated with deployment of various varieties.
