# Supplementary figures and images for: Yellow Rust Epidemics Worldwide Were Caused by Pathogen Races from Divergent Genetic Lineages
Source: Front Plant Sci. 2017 Jun 20;8:1057. doi: 10.3389/fpls.2017.01057 (PMC5477562; doi:10.3389/fpls.2017.01057)

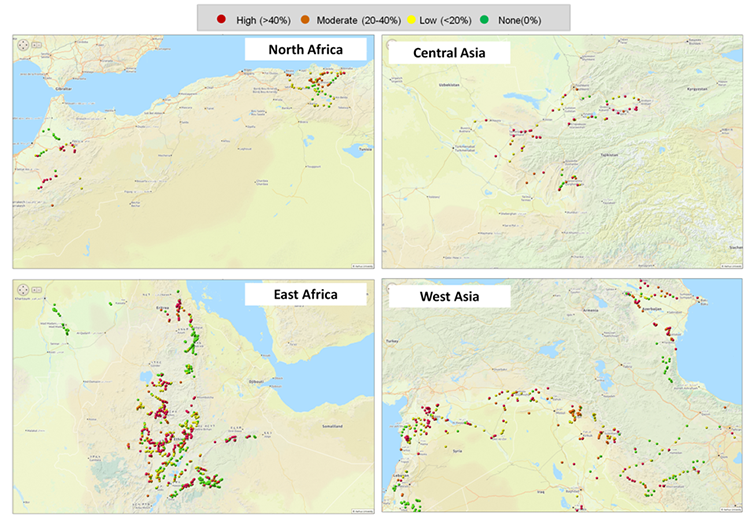

Supplement: Figure S1 — Yellow rust epidemics in across four geographical regions (i.e., central and west Asia and East and North Africa) in the 2010 epidemics season. [file Image1.TIF]
