# Supplementary material for: Yellow Rust Epidemics Worldwide Were Caused by Pathogen Races from Divergent Genetic Lineages
Source: Front Plant Sci. 2017 Jun 20;8:1057. doi: 10.3389/fpls.2017.01057 (PMC5477562; doi:10.3389/fpls.2017.01057)
Supplement: Table S1 — List of collaborators who contributed to the global collection of P. striiformis studied to understand the worldwide pathogen population structure during 2009–2015. [file Table1.doc]

**Table S1. List of collaborators who contributed to the global collection of *P. striiformis* studied to understand the worldwide pathogen population structure during 2009-2015.**

| **Geographical origin** | **Country** | **Collaborating colleagues** | **Contributing institutions to sampling** |
| --- | --- | --- | --- |
|  | Denmark | Mogens S. Hovmollr | Aarhus University, Denmark |
|  |  | Julian Rodriguez-Algaba | Aarhus University, Denmark |
|  |  | Chris K. Sorenson | Aarhus University, Denmark |
|  |  | Lise Jogrsen | Aarhus University, Denmark |
|  |  | Susanne Sindberg | Tystofte Foundation |
|  |  | Stefan Ulrich Ellinger | BASF |
|  |  | Peter Mejnertsen | SEGES |
|  |  | Olav Høegh | Dansk Landbrug Sydhavsøerne |
|  |  | Meta Nielsen | Tystofte Foundation |
|  |  | Martin Ugilt | Landbosyd |
|  |  | Louise Lund | Dansk Landbrug Sydhavsøerne |
|  |  | Lotte Olesen | Nordic Seed |
|  |  | Lars Egelund Olesen | SEGES |
|  |  | Just Bach Andersen | Landbonord |
|  |  | Jeppe Reitan Andersen | Nordic Seed |
|  |  | Janne Hansen | Aarhus University |
|  |  | Henrik Østergaard Nielsen | Djursland Landboforening |
|  |  | Ghita C Nielsen | SEGES |
|  |  | Finn Borum | Sejet Plant Breeding |
|  |  | Ellen Jørgensen | Aarhus University |
|  |  | Anne-Pia Larsen | Aarhus University |
|  | Latvia | Maira Marga | BASF, Latvia |
|  |  | Stefan Ellinger | BASF, Latvia |
|  |  | Oksana Smirnova | BASF, Latvia |
|  | Lithuania | Oksana Smirnova | BASF, Lithuania |
|  | Poland | Danuta-Kurleto | Poland |
|  |  | miroslaw.pojmaj@danko.pl | Poland |
|  |  | Malgorzata Niewinska | Poland |
|  |  | Maria Bogacka | Poland |
|  |  | Pawel Czembor | Poland |
|  |  | Enrique Sánchez-Monge | Limagran, Spain |
|  |  | Isabel Torró | Limagran, Spain |
|  |  | Manuel Infantes | Limagran, Spain |
|  | Sweden | Agnes Jonsson | Jordbruksverket, Sweden |
|  |  | Alf Djurberg | Jordbruksverket, Sweden |
|  |  | Anders Arvidsson | Jordbruksverket, Sweden |
|  |  | Anders Lindgren | Jordbruksverket, Sweden |
|  |  | Anders Karlsson | Jordbruksverket, Sweden |
|  |  | Anna Adler | Jordbruksverket, Sweden |
|  |  | Anna Gerdtsson | Jordbruksverket, Sweden |
|  |  | Anna Pers | Jordbruksverket, Sweden |
|  |  | Caroline Jøngren | Jordbruksverket, Sweden |
|  |  | Cecilia Lerenius | Jordbruksverket, Sweden |
|  |  | Cecilia Söderlind | Jordbruksverket, Sweden |
|  |  | Emmy Johansson | Jordbruksverket, Sweden |
|  |  | Eva Mellquist | Jordbruksverket, Sweden |
|  |  | Fia Birch-Jensen | Jordbruksverket, Sweden |
|  |  | Frida Erlöv | Jordbruksverket, Sweden |
|  |  | GT | Jordbruksverket, Sweden |
|  |  | Gunilla Berg | Jordbruksverket, Sweden |
|  |  | Gunnel Andersson | Jordbruksverket, Sweden |
|  |  | Göran Gustafsson | Jordbruksverket, Sweden |
|  |  | Hans Hedström | Jordbruksverket, Sweden |
|  |  | Jenny Knutsson | Jordbruksverket, Sweden |
|  |  | Joakim Hermansson | Jordbruksverket, Sweden |
|  |  | Johan Andersson | Jordbruksverket, Sweden |
|  |  | Johan Nils | Jordbruksverket, Sweden |
|  |  | Johanna Holmblad | Jordbruksverket, Sweden |
|  |  | Johanna Lindgren | Jordbruksverket, Sweden |
|  |  | Jonathan Sjöström | Jordbruksverket, Sweden |
|  |  | Lars Johansson | Jordbruksverket, Sweden |
|  |  | Lina Norrlund | Jordbruksverket, Sweden |
|  |  | Linnea Aronsson | Jordbruksverket, Sweden |
|  |  | Lisbeth Bergh | Jordbruksverket, Sweden |
|  |  | Louise Adler | Jordbruksverket, Sweden |
|  |  | Louise Aldén | Jordbruksverket, Sweden |
|  |  | M. Thorngren | Jordbruksverket, Sweden |
|  |  | Magnus Sandstrom | Jordbruksverket, Sweden |
|  |  | Mahbubjon Ramatov | Swedish Agricultural University, Uppsala, Sweden |
|  |  | Mariann Wikström | Jordbruksverket, Sweden |
|  |  | Markus Ericsson | Jordbruksverket, Sweden |
|  |  | Mats Selin | Jordbruksverket, Sweden |
|  |  | Mikael Nilsson | Jordbruksverket, Sweden |
|  |  | Niels Wiklund | Jordbruksverket, Sweden |
|  |  | Oskar Björling | Jordbruksverket, Sweden |
|  |  | Oskar Gustafsson | Jordbruksverket, Sweden |
|  |  | Patrik Svanström | Jordbruksverket, Sweden |
|  |  | Robert Dinwiddie | Jordbruksverket, Sweden |
|  |  | Sara Furenhed | Jordbruksverket, Sweden |
|  |  | Sara Wallemyr | Jordbruksverket, Sweden |
| North America | USA | E. Milus | University of Arkansas, USA |
| South America | Argentina | Pablo Campos | INTA, Argentina |
|  | Brazil | Amarilis Barcellos | EMBRAPA, Brazil |
|  |  | André Rosa | EMBRAPA, Brazil |
|  |  | Marcia Chavez | EMBRAPA, Brazil |
|  |  | Ricardo Castro | EMBRAPA, Brazil |
|  | Mexico | Mogen S. Hovmoller | GRRC, Denmark |
|  |  | Julian Rodriguez-Algaba | GRRC, Denmark |
|  | Uruguay | Silivia German | INIA, Uruguay |
| North Africa | Algeria | Chaneze | INRA, Algeria |
|  | Egypt | Atef Shahin | Sakha RC, ARC, Egypt |
|  |  | Essam Abdelhamid | Sakha RC, ARC, Egypt |
|  |  | Mamdouh Asmawi | Sakha RC, ARC, Egypt |
|  |  | Minas Sallam | Sakha RC, ARC, Egypt |
|  |  | Walid E. Orabey | Sakha RC, ARC, Egypt |
|  | Morocco | Amor Yahyaoui | CIMMYT, Mexico |
|  |  | Brahim Ezzahiri | University of Rabat, Morocco |
|  |  | Abdel-Hamid Ramdani | INRA, Morocco |
| West Asia | Azerbaijan | Beyhan Akin | CIMMYT, Turkey |
|  |  | Konul Aslanova | CIMMYT, Turkey |
|  | Iran | Kumarse Nazari | ICARDA, Turkey |
|  |  | Jalal-Kamali | CIMMYT, Iran |
|  |  | Goodarz Najafian | SPII, Iran |
|  |  | Farzad Afshari | SPII, Iran |
|  | Iraq | Abid Al-Hameed Fayadh | Sulaimani University, Iraq |
|  |  | Ahmed Neama Jwad | Sulaimani University, Iraq |
|  |  | Dhia Muhsen Ali | Sulaimani University, Iraq |
|  |  | Emad Al-Maaroof | Sulaimani University, Iraq |
|  |  | Hamid Ali Hadwan | Sulaimani University, Iraq |
|  |  | Hatem Mahmood Hassan | Sulaimani University, Iraq |
|  |  | Hatim Hussien | Sulaimani University, Iraq |
|  |  | Hazha Abdul Karim | Sulaimani University, Iraq |
|  |  | Laith Husain | Sulaimani University, Iraq |
|  | Lebanon | Rola El-Amil | LARI, Lebanon |
|  | Syria | Rola El-Amil | LARI, Lebanon |
|  |  | Maha Al-Ahmed | ICARDA, Turkey |
|  |  | Kumarse Nazari | ICARDA, Turkey |
|  | Turkey | Beyhan Akin | CIMMYT, Turkey |
|  |  | Alexey Morgounov | CIMMYT, Turkey |
|  |  | Zafar Mert | CRIFC, Turkey |
|  |  | Rola El-Amil | LARI, Lebanon |
|  | Yemen | A. Sailan | AREA, Yemen |
|  |  | Musaed Eisa | AREA, Yemen |
|  |  | Mohamed Alsadi | AREA, Yemen |
|  |  | Rashad Basha | AREA, Yemen |
|  |  | Wajeeh Almutawakel | AREA, Yemen |
|  |  | Aref Alshamiri | AREA, Yemen |
| Central Asia | Kazakhstan | Colin Wellings | University of Sydney, Australia |
|  |  | Alma Kohkmetova | Inst Plant Biology & Biotech, Kazakhstan |
|  |  | Gulzat Yessenbekova | Inst Plant Biology & Biotech, Kazakhstan |
|  | Tajikistan | Mahbubjon Ramatov | CIMMYT, Turkey |
|  |  | Bahromiddin Huseinov | CIMMYT, Turkey |
|  |  | Eshonova | CIMMYT, Turkey |
|  |  | Alexey Morgounov | CIMMYT, Turkey |
|  | Uzbekistan | Ram Chandre Sharma | ICARDA, Uzbekistan |
|  |  | Baboev S | ICARDA, Uzbekistan |
|  |  | Zafar Ziyaev | Res Inst Plant Industry, Uzbekistan |
| East Africa | Eritrea | Asmelash Wolday | NARI, Eritrea |
|  | Ethiopia | Dave Hodson | CIMMYT, Ethiopia |
|  |  | Bekele Abeyo | CIMMYT, Ethiopia |
|  |  | Kebede Tadesse | WSU, USA |
|  |  | Geletu Bejiga | ICARDA, Ethiopia |
|  |  | Getaneh Woldeab | EIAR, Ambo PPRC |
|  |  | Netsanet Bacha | EIAR, Ambo PPRC |
|  |  | Zakkie Pretorius | UFS, South Africa |
|  |  | Tizazu | EIAR, Ambo PPRC |
|  |  | Ayele Badebo | CIMMYT, Ethiopia |
|  | Kenya | Ruth Wanyera | KALRO, Njoro, Kenya |
|  |  | Matt Rouse | USDA-ARS, CDL, USA |
|  |  | Kumarse Nazari | ICARDA, Turkey |
|  |  | Mercy Wogah | KALRO, Njoro, Kenya |
|  | Rwanda | Dave Hodson | CIMMYT, Ethiopia |
|  |  | Innocent Habarurema | RAB, Rwanda |
|  |  | Aloys | RAB, Rwanda |
|  |  | Athanase | RAB, Rwanda |
|  |  | Annualite | RAB, Rwanda |
|  |  | Eliane | RAB, Rwanda |
|  |  | Jean Marie | RAB, Rwanda |
|  | Tanzania | Rose Mongi | Uyole RC, Tanzania |
|  |  | Yoseph Alemayehu | CIMMYT, Ethiopia |
|  |  | Salome W. Munissi | SARI, Tanzania |
| South Asia | Afghanistan | Kumarse Nazari | ICARDA, Turkey |
|  |  | Rizvi Javed | ICARDA, Afghanistan |
|  |  | Rick ward | CIMMYT, Pakistan |
|  |  | Rajiv sharma | CIMMYT, Afghanistan |
|  |  | Noorul Haq | ICARDA, Afghanistan |
|  |  | Ahmad Samin Samimy | CIMMYT, Afghanistan |
|  |  | Abdul Rajab | CIMMYT, Afghanistan |
|  |  | Elias Mohmand | CIMMYT, Afghanistan |
|  | Bhutan | Sangay Tshewang | Tsirang Ag Res &Dev sub-center, Min of Ag & Forests |
|  |  | Robert Park | University of Sydney, Australia |
|  |  | Dave Hodson | CIMMYT, Ethiopia |
|  |  | Gordon L. Cisar | Cornell University, USA |
|  |  | Sonam Dorij | Nat Plant Protection Center, Min of Ag & Forests |
|  |  | Legjay | Nat Plant Protection Center, Min of Ag & Forests |
|  |  | Thinley | Nat Plant Protection Center, Min of Ag & Forests |
|  | Nepal | Dhruba B. Thapa | NARC, Nepal |
|  |  | Sarala Lohani/Sharma | NARC, Nepal |
|  |  | Sajid Ali | The University of Agriculture, Peshawar, Pakistan |
|  |  | S. Pradhan | NARC, Nepal |
|  |  | Dave Hodson | CIMMYT, Ethiopia |
|  |  | Basistha Acharya | NARC, Nepal |
|  | Pakistan | Sajid Ali | The University of Agriculture, Peshawar, Pakistan |
|  |  | Rick Ward | CIMMYT, Pakistan |
