# Supplementary material for: Yellow Rust Epidemics Worldwide Were Caused by Pathogen Races from Divergent Genetic Lineages
Source: Front Plant Sci. 2017 Jun 20;8:1057. doi: 10.3389/fpls.2017.01057 (PMC5477562; doi:10.3389/fpls.2017.01057)
Supplement: Table S2 — Details of isolates of P. striiformis virulence phenotyped, SSR genotyped and SCAR genotyped to decipher pathogen's worldwide viulence structure. [file Table2.DOC]

**Table S2. Details of isolates of *P. striiformis* virulence phenotyped, SSR genotyped and SCAR genotyped to decipher pathogen’s worldwide viulence structure.**

| **Lineage** | **Lineage** | **Number of isolates** | | |
| --- | --- | --- | --- | --- |
|  |  | **Race typed** | **SSR genotyped** | **SCAR typed** |
| ***PstS0*** | Brigadier,v4 | 1 | 1 | 1 |
|  | Robigus | 6 | 4 | 4 |
|  | Solstice/Oakley | 14 | 11 | 8 |
|  | Tulsa | 32 | 13 | 13 |
| ***PstS1*** | PstS1,v10,v24,v27 | 2 | 2 | 2 |
|  | PstS1,v17 | 2 | - | - |
|  | PstS1,v17,v27 | 2 | - | - |
|  | PstS1,v3,v17,v27 | 1 | - | 1 |
|  | PstS1,v3,v17,v27,v32 | 2 | - | 2 |
| ***PstS2*** | Pst2,v1,v27 | 43 | 11 | 43 |
|  | PstS2 | 35 | 17 | 15 |
|  | PstS2,v1 | 4 | 4 | 4 |
|  | PstS2,v10,v24 | 6 | 2 | 2 |
|  | PstS2,v10,v24,v27 | 1 | - | 1 |
|  | PstS2,v27 | 80 | 42 | 31 |
|  | PstS2,v3 | 9 | - | 8 |
|  | PstS2,v3,v10,v24,27 | 1 | - | 1 |
|  | PstS2,v3,v27 | 9 | 1 | 6 |
| ***PstS3*** | PstS3 | 6 | 6 | 3 |
|  | PstS3,v10,v24 | 1 | - | - |
|  | PstS3(-) | 2 | - | - |
| ***PstS4*** | PstS4 | 84 | 35 | 40 |
| ***PstS5*** | PstS5 | 27 | 25 | 14 |
|  | PstS5,v17 | 3 | 3 | 1 |
| ***PstS6*** | PstS6 | 41 | 21 | 19 |
| ***PstS7*** | PstS7 | 102 | 33 | 3 |
| ***PstS8*** | PstS8 | 152 | 42 | 9 |
| ***PstS9*** | PstS9 | 11 | 10 | 1 |
|  | PstS9,v17 | 14 | 14 | 2 |
| ***PstS10*** | PstS10 | 49 | 1 | - |
| **Other** | Other | 145 | 75 | 39 |
|  | Total | 887 | 373 | 273 |
