# Supplementary material for: Yellow Rust Epidemics Worldwide Were Caused by Pathogen Races from Divergent Genetic Lineages
Source: Front Plant Sci. 2017 Jun 20;8:1057. doi: 10.3389/fpls.2017.01057 (PMC5477562; doi:10.3389/fpls.2017.01057)
Supplement: Table S4 — Percentage (%) of different virulences in worldwide P. striiformis population samplescollected during 2009–2015. [file Table4.DOC]

**Table S4. Percentage (%) of different virulences in worldwide *P. striiformis* population samplescollected during 2009-2015.**

| **Virulence** | **Europe** | **North America** | **South America** | **North Africa** | **West Asia** | **Central Asia** | **East Africa** | **South Asia** | **Overall population** |
| --- | --- | --- | --- | --- | --- | --- | --- | --- | --- |
| ***Vr1*** | 69 | 13 | 39 | 24 | 4 | 100 | 60 | 66 | 60 |
| ***Vr2*** | 92 | 100 | 89 | 92 | 94 | 100 | 99 | 81 | 93 |
| ***Vr3*** | 76 | 50 | 89 | 24 | 0 | 95 | 15 | 21 | 54 |
| ***Vr4*** | 42 | 0 | 89 | 24 | 0 | 95 | 0 | 44 | 36 |
| ***Vr5*** | 0 | 0 | 0 | 0 | 0 | 0 | 0 | 0 | 0 |
| ***Vr6*** | 97 | 100 | 89 | 100 | 100 | 100 | 99 | 90 | 97 |
| ***Vr7*** | 87 | 100 | 89 | 100 | 100 | 5 | 99 | 90 | 86 |
| ***Vr8*** | 57 | 100 | 0 | 76 | 99 | 4 | 73 | 64 | 60 |
| ***Vr9*** | 73 | 100 | 29 | 92 | 93 | 96 | 91 | 60 | 77 |
| ***Vr10*** | 18 | 0 | 0 | 0 | 7 | 0 | 10 | 0 | 12 |
| ***Vr15*** | 0 | 0 | 0 | 0 | 0 | 0 | 0 | 0 | 0 |
| ***Vr17*** | 68 | 100 | 29 | 24 | 0 | 30 | 28 | 60 | 50 |
| ***Vr24*** | 18 | 0 | 0 | 0 | 7 | 0 | 9 | 0 | 12 |
| ***Vr25*** | 76 | 100 | 89 | 92 | 93 | 96 | 69 | 30 | 75 |
| ***Vr27*** | 0 | 75 | 29 | 68 | 62 | 46 | 77 | 53 | 30 |
| ***Vr32*** | 76 | 25 | 0 | 24 | 0 | 95 | 0 | 30 | 49 |
| ***VrSp*** | 31 | 0 | 0 | 24 | 0 | 0 | 0 | 12 | 18 |
| ***VrAvS*** | 81 | 100 | 89 | 100 | 100 | 100 | 98 | 99 | 89 |
| ***VrAmb*** | 53 | 13 | 0 | 22 | 0 | 95 | 1 | 18 | 36 |
| **No. of isolates tested** | 463 | 8 | 28 | 37 | 81 | 56 | 137 | 77 | 887 |

Figures and symbols designate virulence and avirulence(-) corresponding to yellow rust resistance genes: *Yr1, Yr2, Yr3, Yr4, Yr5, Yr6, Yr7, Yr8, Yr9, Yr10, Yr15, Yr17, Yr24, Yr25, Yr27, Yr32*, and the resistance specificity of Spalding Prolific (Sp), Avocet S (AvS) and Ambition (Amb), respectively.
