# Supplementary material for: Yellow Rust Epidemics Worldwide Were Caused by Pathogen Races from Divergent Genetic Lineages
Source: Front Plant Sci. 2017 Jun 20;8:1057. doi: 10.3389/fpls.2017.01057 (PMC5477562; doi:10.3389/fpls.2017.01057)
Supplement: Table S5 — Worldwide prevalence of P. striiformis racesacross eight geographical regions during wheat growing seasons in the period 2009–2015. [file Table5.DOC]

**Table S5. Worldwide prevalence of *P. striiformis* racesacross eight geographical regions during wheat growing seasons in the period 2009-2015.**

| **Pathotype code full** | **Race Name**  **(if applicable)** | **Europe** | **North America** | **South America** | **North Africa** | **West Asia** | **Central Asia** | **East Africa** | **South Asia** | **Overall Population** |
| --- | --- | --- | --- | --- | --- | --- | --- | --- | --- | --- |
| 1,2,3,-,-,6,7,8,9,-,-,17,-,25,-,32,-,AvS,Amb | PstS8 | 32.83 | 0.00 | 0.00 | 0.00 | 0.00 | 0.00 | 0.00 | 0.00 | 17.14 |
| 1,2,3,4,-,6,7,-,9,-,-,17,-,25,-,32,Sp,AvS,Amb | PstS7 | 20.30 | 0.00 | 0.00 | 21.62 | 0.00 | 0.00 | 0.00 | 0.00 | 11.39 |
| -,2,-,-,-,6,7,8,-,10,-,-,24,-,-,-,-,-,- | PstS4 | 18.14 | 0.00 | 0.00 | 0.00 | 0.00 | 0.00 | 0.00 | 0.00 | 9.47 |
| 1,2,3,4,-,6,7,-,9,-,-,17,-,25,-,32,Sp,AvS,- | PstS10 | 10.37 | 0.00 | 0.00 | 2.70 | 0.00 | 0.00 | 0.00 | 0.00 | 5.52 |
| -,-,3,4,-,6,-,-,-,-,-,-,-,25,-,32,-,AvS,- | Tulsa | 6.91 | 0.00 | 0.00 | 0.00 | 0.00 | 0.00 | 0.00 | 0.00 | 3.61 |
| -,2,-,-,-,6,7,8,9,-,-,-,-,-,-,-,-,AvS,- | PstS11 | 4.32 | 0.00 | 0.00 | 0.00 | 0.00 | 0.00 | 0.00 | 0.00 | 2.25 |
| 1,2,3,4,-,6,-,-,9,-,-,17,-,25,-,32,-,AvS,- | Solstice/Oakley | 3.02 | 0.00 | 0.00 | 0.00 | 0.00 | 0.00 | 0.00 | 0.00 | 1.58 |
| 1,2,3,4,-,-,-,-,9,-,-,17,-,25,-,32,-,AvS,- | Robigus | 1.30 | 0.00 | 0.00 | 0.00 | 0.00 | 0.00 | 0.00 | 0.00 | 0.68 |
| -,-,-,-,-,-,-,-,-,-,-,-,-,-,-,-,-,-,- | Non-wheat | 0.86 | 0.00 | 0.00 | 0.00 | 0.00 | 0.00 | 0.00 | 0.00 | 0.45 |
| 1,2,3,4,-,-,-,-,9,-,-,17,-,25,-,-,-,AvS,- | Brigadier,v4 | 0.22 | 0.00 | 0.00 | 0.00 | 0.00 | 0.00 | 0.00 | 0.00 | 0.11 |
| -,-,-,-,-,6,7,8,-,-,-,-,-,-,-,-,-,-,- | PstS3(-) | 0.22 | 0.00 | 0.00 | 0.00 | 0.00 | 0.00 | 0.00 | 1.30 | 0.23 |
| -,2,3,-,-,6,7,8,-,-,-,-,-,25,-,32,-,AvS,- | Not-assigned | 0.43 | 0.00 | 0.00 | 0.00 | 0.00 | 0.00 | 0.00 | 0.00 | 0.23 |
| -,2,-,-,-,6,7,8,-,-,-,-,-,25,-,-,-,AvS,- | Not-assigned | 0.22 | 0.00 | 0.00 | 0.00 | 0.00 | 0.00 | 0.73 | 0.00 | 0.23 |
| 1,2,3,-,-,-,-,-,9,-,-,17,-,25,-,-,-,AvS,- | Not-assigned | 0.22 | 0.00 | 0.00 | 0.00 | 0.00 | 0.00 | 0.00 | 1.30 | 0.23 |
| -,2,-,-,-,6,7,8,-,-,-,-,-,25,-,32,-,AvS,- | Not-assigned | 0.22 | 0.00 | 0.00 | 0.00 | 0.00 | 0.00 | 0.00 | 0.00 | 0.11 |
| 1,-,3,4,-,6,-,-,-,-,-,-,-,25,-,32,-,AvS,- | Not-assigned | 0.22 | 0.00 | 0.00 | 0.00 | 0.00 | 0.00 | 0.00 | 0.00 | 0.11 |
| 1,2,3,-,-,6,-,-,9,-,-,17,-,25,-,32,-,AvS,- | Not-assigned | 0.22 | 0.00 | 0.00 | 0.00 | 0.00 | 0.00 | 0.00 | 0.00 | 0.11 |
| -,2,-,-,-,6,7,8,9,-,-,17,-,25,-,-,-,AvS,- | PstS1,v17 | 0.00 | 25.00 | 0.00 | 0.00 | 0.00 | 0.00 | 0.00 | 0.00 | 0.23 |
| -,2,-,-,-,6,7,8,9,-,-,17,-,25,27,-,-,AvS,- | PstS1,v17,v27 | 0.00 | 25.00 | 0.00 | 0.00 | 0.00 | 0.00 | 0.00 | 0.00 | 0.23 |
| -,2,3,-,-,6,7,8,9,-,-,17,-,25,27,32,-,AvS,- | PstS1,v3,v17,v27,v32 | 0.00 | 25.00 | 0.00 | 0.00 | 0.00 | 0.00 | 0.00 | 0.00 | 0.23 |
| -,2,3,-,-,6,7,8,9,-,-,17,-,25,27,-,-,AvS,- | PstS1,v3,v17,v27 | 0.00 | 12.50 | 0.00 | 0.00 | 0.00 | 0.00 | 0.00 | 0.00 | 0.11 |
| 1,2,3,-,-,6,7,8,9,-,-,17,-,25,27,-,-,AvS,Amb | Not-assigned | 0.00 | 12.50 | 0.00 | 0.00 | 0.00 | 0.00 | 0.00 | 0.00 | 0.11 |
| -,2,3,4,-,6,7,-,-,-,-,-,-,25,-,-,-,AvS,- | Not-assigned | 0.00 | 0.00 | 60.71 | 0.00 | 0.00 | 0.00 | 0.00 | 0.00 | 1.92 |
| 1,2,3,4,-,6,7,-,9,-,-,17,-,25,27,-,-,AvS,- | Not-assigned | 0.00 | 0.00 | 28.57 | 0.00 | 0.00 | 0.00 | 0.00 | 0.00 | 0.90 |
| 1,-,-,-,-,-,-,-,-,-,-,-,-,-,-,-,-,-,- | Not-assigned | 0.00 | 0.00 | 10.71 | 0.00 | 0.00 | 0.00 | 0.00 | 0.00 | 0.34 |
| -,2,-,-,-,6,7,8,9,-,-,-,-,25,27,-,-,AvS,- | PstS2,v27 | 0.00 | 0.00 | 0.00 | 67.57 | 56.79 | 0.00 | 6.57 | 0.00 | 8.91 |
| -,-,-,-,-,6,7,8,-,-,-,-,-,-,-,-,-,AvS,- | PstS3 | 0.00 | 0.00 | 0.00 | 8.11 | 3.70 | 0.00 | 0.00 | 0.00 | 0.68 |
| -,2,-,-,-,6,7,8,9,-,-,-,-,25,-,-,-,AvS,- | PstS2 | 0.00 | 0.00 | 0.00 | 0.00 | 28.40 | 0.00 | 6.57 | 3.90 | 3.95 |
| -,2,-,-,-,6,7,8,9,10,-,-,24,25,-,-,-,AvS,- | PstS2,v10,v24 | 0.00 | 0.00 | 0.00 | 0.00 | 4.94 | 0.00 | 2.92 | 0.00 | 0.90 |
| 1,2,-,-,-,6,7,8,9,-,-,-,-,25,27,-,-,AvS,- | Pst2,v1,v27 | 0.00 | 0.00 | 0.00 | 0.00 | 1.23 | 0.00 | 30.66 | 0.00 | 4.85 |
| 1,2,-,-,-,6,7,8,9,-,-,-,-,25,-,-,-,AvS,- | PstS2,v1 | 0.00 | 0.00 | 0.00 | 0.00 | 1.23 | 0.00 | 2.19 | 0.00 | 0.45 |
| -,-,-,-,-,6,7,8,-,10,-,-,24,-,-,-,-,AvS,- | PstS3,v10,v24 | 0.00 | 0.00 | 0.00 | 0.00 | 1.23 | 0.00 | 0.00 | 0.00 | 0.11 |
| -,-,-,-,-,6,7,8,-,10,-,-,24,-,27,-,-,AvS,- | Not-assigned | 0.00 | 0.00 | 0.00 | 0.00 | 1.23 | 0.00 | 0.00 | 0.00 | 0.11 |
| 1,2,-,-,-,6,7,-,-,-,-,-,-,-,-,-,-,AvS,- | Not-assigned | 0.00 | 0.00 | 0.00 | 0.00 | 1.23 | 0.00 | 0.00 | 0.00 | 0.11 |
| 1,2,3,4,-,6,-,-,9,-,-,-,-,25,-,32,-,AvS,Amb | PstS5 | 0.00 | 0.00 | 0.00 | 0.00 | 0.00 | 50.00 | 0.00 | 0.00 | 3.16 |
| 1,2,3,4,-,6,-,-,9,-,-,17,-,25,27,32,-,AvS,Amb | PstS9,v17 | 0.00 | 0.00 | 0.00 | 0.00 | 0.00 | 25.00 | 0.00 | 0.00 | 1.58 |
| 1,2,3,4,-,6,-,-,9,-,-,-,-,25,27,32,-,AvS,Amb | PstS9 | 0.00 | 0.00 | 0.00 | 0.00 | 0.00 | 16.07 | 0.00 | 1.30 | 1.13 |
| 1,2,3,4,-,6,-,-,9,-,-,17,-,25,-,32,-,AvS,Amb | PstS5,v17 | 0.00 | 0.00 | 0.00 | 0.00 | 0.00 | 3.57 | 0.00 | 1.30 | 0.34 |
| 1,2,-,-,-,6,7,-,9,-,-,17,-,-,27,-,-,AvS,- | PstS6 | 0.00 | 0.00 | 0.00 | 0.00 | 0.00 | 1.79 | 25.55 | 6.49 | 4.62 |
| 1,2,-,-,-,6,7,8,-,-,-,-,-,-,27,-,-,AvS,- | Not-assigned | 0.00 | 0.00 | 0.00 | 0.00 | 0.00 | 1.79 | 0.00 | 0.00 | 0.11 |
| 1,2,-,-,-,6,7,8,-,-,-,-,-,25,27,-,-,AvS,- | Not-assigned | 0.00 | 0.00 | 0.00 | 0.00 | 0.00 | 1.79 | 0.00 | 0.00 | 0.11 |
| -,2,3,-,-,6,7,8,9,-,-,-,-,25,-,-,-,AvS,- | PstS2,v3 | 0.00 | 0.00 | 0.00 | 0.00 | 0.00 | 0.00 | 6.57 | 0.00 | 1.01 |
| -,2,3,-,-,6,7,8,9,-,-,-,-,25,27,-,-,AvS,- | PstS2,v3,v27 | 0.00 | 0.00 | 0.00 | 0.00 | 0.00 | 0.00 | 4.38 | 3.90 | 1.01 |
| -,2,-,-,-,6,7,8,-,10,-,-,24,-,27,-,-,AvS,- | Not-assigned | 0.00 | 0.00 | 0.00 | 0.00 | 0.00 | 0.00 | 2.19 | 0.00 | 0.34 |
| -,2,-,-,-,6,7,8,9,10,-,-,24,25,27,-,-,AvS,- | PstS1,v10,v24,v27 | 0.00 | 0.00 | 0.00 | 0.00 | 0.00 | 0.00 | 2.19 | 0.00 | 0.34 |
| -,2,-,-,-,6,7,8,-,-,-,-,-,25,27,-,-,AvS,- | Not-assigned | 0.00 | 0.00 | 0.00 | 0.00 | 0.00 | 0.00 | 1.46 | 0.00 | 0.23 |
| -,-,-,-,-,-,-,-,-,-,-,-,-,-,27,-,-,-,- | Not-assigned | 0.00 | 0.00 | 0.00 | 0.00 | 0.00 | 0.00 | 0.73 | 0.00 | 0.11 |
| -,-,-,-,-,6,7,8,9,10,-,-,24,-,-,-,-,-,- | Not-assigned | 0.00 | 0.00 | 0.00 | 0.00 | 0.00 | 0.00 | 0.73 | 0.00 | 0.11 |
| -,2,-,-,-,6,7,8,-,-,-,17,-,-,-,-,-,AvS,- | Not-assigned | 0.00 | 0.00 | 0.00 | 0.00 | 0.00 | 0.00 | 0.73 | 0.00 | 0.11 |
| -,2,-,-,-,6,7,8,-,10,-,-,-,-,-,-,-,-,- | Not-assigned | 0.00 | 0.00 | 0.00 | 0.00 | 0.00 | 0.00 | 2.19 | 0.00 | 0.11 |
| -,2,3,-,-,6,7,8,-,-,-,-,-,25,27,-,-,AvS,- | PstS2,v3,v27 | 0.00 | 0.00 | 0.00 | 0.00 | 0.00 | 0.00 | 0.73 | 0.00 | 0.11 |
| -,2,3,-,-,6,7,8,-,10,-,-,24,25,27,-,-,AvS,- | Not-assigned | 0.00 | 0.00 | 0.00 | 0.00 | 0.00 | 0.00 | 0.73 | 0.00 | 0.11 |
| -,2,3,-,-,6,7,8,9,10,-,-,24,25,27,-,-,AvS,- | PstS2,v3,v10,v24,v27 | 0.00 | 0.00 | 0.00 | 0.00 | 0.00 | 0.00 | 0.73 | 0.00 | 0.11 |
| 1,2,-,-,-,6,7,-,-,-,-,17,-,25,-,-,-,AvS,- | Not-assigned | 0.00 | 0.00 | 0.00 | 0.00 | 0.00 | 0.00 | 0.73 | 0.00 | 0.11 |
| 1,2,-,-,-,6,7,8,-,-,-,17,-,25,-,-,-,AvS,- | Not-assigned | 0.00 | 0.00 | 0.00 | 0.00 | 0.00 | 0.00 | 0.73 | 0.00 | 0.11 |
| -,2,-,4,-,6,7,8,-,-,-,17,-,-,27,32,-,AvS,- | Not-assigned | 0.00 | 0.00 | 0.00 | 0.00 | 0.00 | 0.00 | 0.00 | 12.99 | 1.13 |
| 1,2,3,4,-,6,7,-,9,-,-,17,-,25,-,32,Sp,AvS,Amb | Not-assigned | 0.00 | 0.00 | 0.00 | 0.00 | 0.00 | 0.00 | 0.00 | 10.39 | 0.90 |
| 1,2,-,-,-,6,7,8,-,-,-,17,-,-,-,-,-,AvS,- | Not-assigned | 0.00 | 0.00 | 0.00 | 0.00 | 0.00 | 0.00 | 0.00 | 7.79 | 0.68 |
| 1,-,-,-,-,6,7,-,9,-,-,-,-,-,27,-,-,AvS,- | Not-assigned | 0.00 | 0.00 | 0.00 | 0.00 | 0.00 | 0.00 | 0.00 | 6.49 | 0.56 |
| 1,2,-,-,-,6,7,8,9,-,-,17,-,-,27,-,-,AvS,- | Not-assigned | 0.00 | 0.00 | 0.00 | 0.00 | 0.00 | 0.00 | 0.00 | 5.19 | 0.45 |
| 1,2,-,4,-,6,7,8,9,-,-,17,-,-,27,-,-,AvS,- | Not-assigned | 0.00 | 0.00 | 0.00 | 0.00 | 0.00 | 0.00 | 0.00 | 5.19 | 0.45 |
| -,-,-,-,-,-,-,8,-,-,-,-,-,-,-,-,-,AvS,- | Not-assigned | 0.00 | 0.00 | 0.00 | 0.00 | 0.00 | 0.00 | 0.00 | 3.90 | 0.34 |
| 1,-,-,-,-,-,7,-,9,-,-,-,-,-,27,-,-,AvS,- | Not-assigned | 0.00 | 0.00 | 0.00 | 0.00 | 0.00 | 0.00 | 0.00 | 3.90 | 0.34 |
| 1,2,-,4,-,6,7,8,-,-,-,-,-,-,-,-,-,AvS,- | Not-assigned | 0.00 | 0.00 | 0.00 | 0.00 | 0.00 | 0.00 | 0.00 | 3.90 | 0.34 |
| 1,2,-,4,-,6,7,8,-,-,-,17,-,-,-,-,-,AvS,- | Not-assigned | 0.00 | 0.00 | 0.00 | 0.00 | 0.00 | 0.00 | 0.00 | 2.60 | 0.23 |
| -,2,3,-,-,6,7,8,9,-,-,17,-,25,-,-,-,AvS,Amb | Not-assigned | 0.00 | 0.00 | 0.00 | 0.00 | 0.00 | 0.00 | 0.00 | 1.30 | 0.11 |
| -,2,-,-,-,6,-,8,-,-,-,-,-,-,-,-,Sp,AvS,- | Not-assigned | 0.00 | 0.00 | 0.00 | 0.00 | 0.00 | 0.00 | 0.00 | 1.30 | 0.11 |
| -,2,-,-,-,6,7,-,-,-,-,-,-,25,-,-,-,AvS,- | Not-assigned | 0.00 | 0.00 | 0.00 | 0.00 | 0.00 | 0.00 | 0.00 | 1.30 | 0.11 |
| -,2,-,-,-,6,7,-,-,-,-,17,-,25,-,-,-,AvS,Amb | Not-assigned | 0.00 | 0.00 | 0.00 | 0.00 | 0.00 | 0.00 | 0.00 | 1.30 | 0.11 |
| -,2,-,4,-,6,7,8,9,-,-,17,-,-,27,-,-,AvS,- | Not-assigned | 0.00 | 0.00 | 0.00 | 0.00 | 0.00 | 0.00 | 0.00 | 1.30 | 0.11 |
| -,2,-,4,-,6,7,8,9,-,-,17,-,-,27,32,-,AvS,- | Not-assigned | 0.00 | 0.00 | 0.00 | 0.00 | 0.00 | 0.00 | 0.00 | 1.30 | 0.11 |
| 1,-,-,-,-,-,7,-,9,-,-,-,-,-,-,-,-,AvS,- | Not-assigned | 0.00 | 0.00 | 0.00 | 0.00 | 0.00 | 0.00 | 0.00 | 1.30 | 0.11 |
| 1,-,-,-,-,6,7,8,-,-,-,-,-,-,-,-,-,AvS,- | Not-assigned | 0.00 | 0.00 | 0.00 | 0.00 | 0.00 | 0.00 | 0.00 | 1.30 | 0.11 |
| 1,-,-,-,-,6,7,8,9,-,-,-,-,-,27,-,-,AvS,- | Not-assigned | 0.00 | 0.00 | 0.00 | 0.00 | 0.00 | 0.00 | 0.00 | 1.30 | 0.11 |
| 1,2,-,-,-,6,7,-,-,-,-,-,-,25,-,-,-,AvS,- | Not-assigned | 0.00 | 0.00 | 0.00 | 0.00 | 0.00 | 0.00 | 0.00 | 1.30 | 0.11 |
| 1,2,-,-,-,6,7,8,9,-,-,-,-,25,27,-,-,AvS,- | Not-assigned | 0.00 | 0.00 | 0.00 | 0.00 | 0.00 | 0.00 | 0.00 | 1.30 | 0.11 |
| 1,2,-,4,-,6,7,8,-,-,-,17,-,-,27,-,-,AvS,Amb | Not-assigned | 0.00 | 0.00 | 0.00 | 0.00 | 0.00 | 0.00 | 0.00 | 1.30 | 0.11 |
| 1,2,-,4,-,6,7,8,9,-,-,17,-,-,27,32,-,AvS,- | Not-assigned | 0.00 | 0.00 | 0.00 | 0.00 | 0.00 | 0.00 | 0.00 | 1.30 | 0.11 |
| 1,2,3,4,-,6,-,8,9,-,-,-,-,25,-,32,-,AvS,Amb | Not-assigned | 0.00 | 0.00 | 0.00 | 0.00 | 0.00 | 0.00 | 0.00 | 1.30 | 0.11 |
| Number of isolates tested | | 463 | 8 | 28 | 37 | 81 | 56 | 137 | 77 | 887 |
